# Neuroleptic Malignant Syndrome From Oxcarbazepine and Topiramate Withdrawal: An Unusual Case

**DOI:** 10.7759/cureus.29992

**Published:** 2022-10-06

**Authors:** Son Dang, Sachidanand Peteru, Mohammad Raja, Arsen Askandaryan, Grace Vallejo

**Affiliations:** 1 Department of Psychiatry, Jamaica Hospital Medical Center, New York, USA; 2 Department of Internal Medicine, Jamaica Hospital Medical Center, New York, USA

**Keywords:** medication-induced nms, oxcarbazepine, topiramate, antipsychotics, neuroleptic malignant syndrome

## Abstract

Neuroleptic malignant syndrome (NMS) is a rare, but fatal adverse reaction that is most commonly seen with typical antipsychotic medications. However, NMS can also be triggered by other dopamine-modulating agents that physicians are unlikely aware of, leading to being underdiagnosed or precluding early recognition of the syndrome. We describe a case involving a 20-year-old male who presented to the emergency department with altered mental status and failure to thrive. On admission, he subsequently developed an insidious onset of muscle rigidity and autonomic instability, and laboratory work-up was significant for leukocytosis, transaminitis, and elevations in creatinine phosphokinase, lactate, and C-reactive protein. After a battery of negative diagnostic tests, his clinical features fulfilled the NMS criteria by a diagnosis of exclusion, even in the absence of any antipsychotic regimen or dopaminergic medications. Management with dantrolene, amantadine, and aggressive fluid therapy provided a gradual return of the patient’s baseline mentation along with normalization in laboratory assessments. In this novel case of NMS, we suspect oxcarbazepine and topiramate withdrawal as possible attributing factors for the patient’s presentation. This article emphasizes the need for hypervigilance in future cases with high suspicion of NMS, in addition to raising a broader clinical awareness of other potential etiologies of NMS that are not restricted to only antipsychotic medications. We further discuss a review of the pathophysiology, various etiologies, clinical features, diagnostic criteria, treatment plans, and complications of NMS.

## Introduction

Neuroleptic malignant syndrome (NMS) is a rare, but potentially fatal idiosyncratic reaction associated with the use of dopamine-modulating pharmacologic agents in a dose-independent fashion, most noticeably linked to the administration of high-potency, first-generation typical antipsychotics. The incidence rate of NMS is estimated between 0.02% and 3% from various regional studies, however, clinicians should continue to be vigilant due to the prevalent usage and plentiful variations of dopamine-modulating medications, as well as their increasing implications in the management of common physical and mental disorders. Considering that NMS is a strikingly lethal adverse reaction with an estimated mortality ranging from 7% to 15%, an early recognition, diagnosis, and aggressive treatment plan is imperative to a patient’s prognosis [1]. This article aims to review the pathophysiology, etiologies, and clinical presentation of NMS in addition to describing an intricate case of NMS resulting from an unanticipated stoppage of oxcarbazepine and topiramate in the absence of direct antidopaminergic modulators.

## Case presentation

A 20-year-old African American male with an extensive history of autistic spectrum disorder with behavior disturbances, generalized anxiety disorder (GAD), disruptive mood dysregulation disorder (DMDD), spastic cerebral palsy with left upper extremity monoplegia, and class III morbid obesity was brought into the emergency department (ED) by his parents due to gradual worsening aggressive behavior and poor oral intake for a duration of eight days. Within the most recent two days, the patient also suffered from insomnia and intermittent nausea. During that time, his parents attributed his symptoms to the possibility of depression and clarified the patient’s non-compliance to his psychiatric medications since the onset of poor feeding. His psychiatric medications included topiramate 200 mg at bedtime for weight loss and insomnia, oxcarbazepine 600 mg BID for the off-label management of DMDD, lorazepam 1 mg TID, and clonidine 0.2 mg BID for GAD.

**Figure 1:**
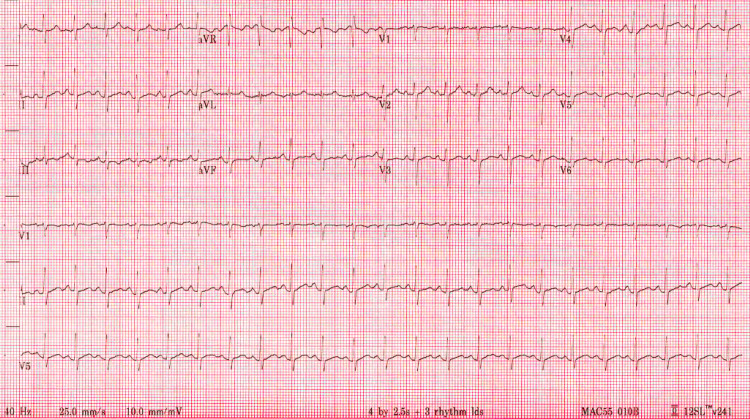
Electrocardiogram on Admission Electrocardiogram showing sinus tachycardia at an approximate heart rate of 150 beats per minute. The correct QT interval by the Bazett formula is 379 milliseconds (within the normal range of 350-450 ms for adult males).

Initial valuation in the ED was limited due to the patient being non-verbal with altered mental status, although his parents reported being talkative at baseline. Initial vital signs were significant for hypotension (blood pressure 70/46 mmHg) and tachycardia (heart rate 148 bpm); however, he was afebrile at 98 F (36.7 C). On physical examination, the patient appeared verbally mute with obese body habitus. Bilateral upper extremities had a limited passive range of motion with difficulty differentiating between stiffness and intentional resistance. Fluid resuscitation using normal saline was initiated with effective hemodynamic stabilization, and the patient was admitted presumptively for the management of dehydration with secondary acute kidney injury while awaiting the remainder of his initial workup. Upon admission, the patient spiked a fever of 102.7 F (39.3 C) in the presence of persistent tachycardia, prompting sepsis protocol activation and additional tests sent for a fever of unknown origin. Initial laboratory findings that were significant can be found in Table 1, along with the patient's electrocardiogram on admission in Figure 1.

**Table 1:** Significant Labs and Imaging on Admission Table showing the initial labs upon admission. The content in bold represents significant variations in value from the normal reference range.

Significant Labs and Imaging on Admission	
Complete blood cell count	White blood cells 13,600/microliter
	Neutrophils 76.5%
	Lymphocyte 13.8%
	Monocyte 8.5%
	Eosinophil 0%
	Basophils 1.2%
	Red blood cells 6.23x10^9^/microliter
	Hemoglobin 16.5 g/dL
	Hematocrit 52.4%
	Mean corpuscular 84.1 fL
	Red cell distribution width 15.3%
	Platelet 200,000/microliter
Complete Metabolic Panel	Glucose 156 mg/dL
	Blood urea nitrogen 30 mg/dL
	Creatinine 2.0 mg/dL
	Sodium 148 mEq/L
	Potassium 4.2 mEq/L
	Chloride 115 mEq/L
	Carbon dioxide 18 mEq/L
	Calcium 9.4 mg/dL
	Anion gap 15 mEq/L
	Magnesium 2.2 mg/dL
	Total protein 8.2 g/dL
	Albumin 4.5 g/dL
	Total bilirubin 0.9 mg/dL
	Alanine transaminase 146 U/L
	Aspartate transaminase 225 U/L
	Alkaline phosphatase 89 U/L
Lactate	2.25 mg/dL
C-reactive protein	3.1 mg/dL
Procalcitonin	0.39 ng/mL
Creatinine phosphokinase	15,428 U/L
Urinalysis	Dark yellow and turbid
	Specific gravity > 1.030
	pH 6.0
	Protein > 600 mg/dL
	Urinary glucose: 30 mg/dL
	Ketones: negative
	Bilirubin: small
	Blood: moderate
	Nitrite: negative
	Leukocytes: negative
	White blood cells: 6/hpf
	Red blood cells: 15/hpf
	Bacteria: few
Chest X-ray	Negative for abnormalities

Simultaneously, a psychiatric consultation-liaison (C&L) was consulted for control of agitation due to incidences of behavioral aggression and intravenous line pulling. Amid clinical suspicion, C&L decided to not initiate any psychotropic medications while awaiting diagnostic workup to rule out NMS and prevent exacerbating symptoms. Intramuscular or intravenous lorazepam was recommended at the time for the management of aggressive agitation. Infectious disease consultation recommended a lumbar puncture to rule out septic meningitis and encephalitis with empirical treatment using ceftriaxone, acyclovir, vancomycin, and dexamethasone. Despite increasing fluid management along with cooling blankets and non-steroidal anti-inflammatory drugs (NSAIDs) administration, the patient’s fever was unwavering and the interval creatine phosphokinase (CPK) assays elevated to a peak of 39,771 U/L. In the setting of persistent fever and worsening rhabdomyolysis, an ICU upgrade was necessitated for closer monitoring. CT of the head, chest, and abdomen pelvis demonstrated no acute pathologic changes. MRI of the head could not be obtained due to the patient’s body habitus and weight (372-pounds/167.8 kilograms) exceeding the facility’s 350-pound scanner limitation. An electroencephalogram was obtained and ruled out any seizure activities or status epilepticus. Finalized results from cerebrospinal fluid studies ultimately ruled out infectious meningoencephalitis with the discontinuation of antibiotics and antiviral therapy. Anesthesiology consultation for malignant hyperthermia was unlikely due to the lack of exposure to any anesthetics or neuromuscular paralytics.

**Figure 2:**
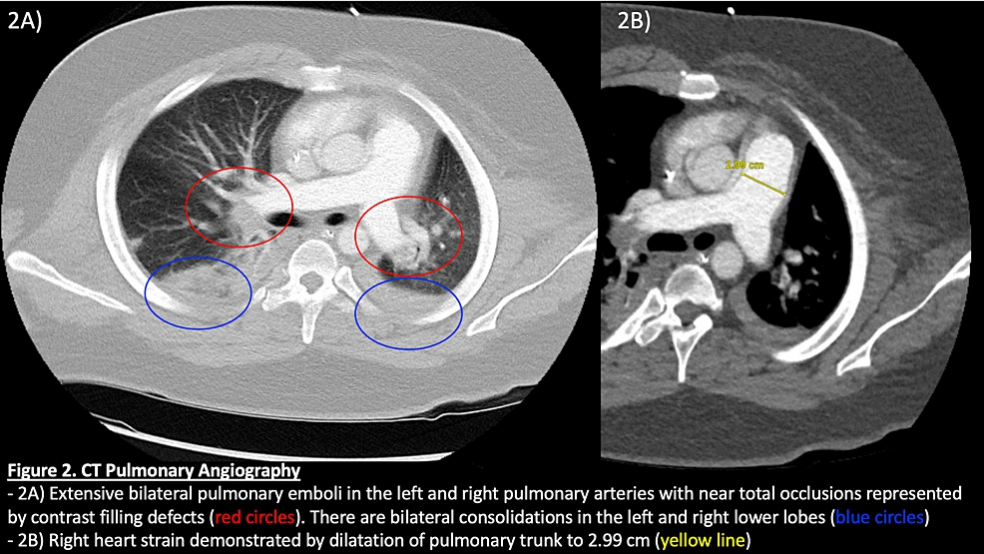
CT Pulmonary Angiography of Bilateral Pulmonary Emboli - 2A) Extensive bilateral pulmonary emboli in the left and right pulmonary arteries with near total occlusions represented by contrast filling defects (red circles). Bilateral consolidations can also be seen in the left and right lower lobes (blue circles). - 2B) Evidence of right-sided heart strain is demonstrated by dilatation of the pulmonary trunk to 2.99 cm (yellow line).

Subsequently, due to the unyielding results of a robust diagnostic process, the presumptive neuroleptic malignant syndrome was considered a diagnosis of exclusion. The patient received dantrolene 150 mg TID for three days in addition to C&L's recommendation of amantadine 100 mg BID and aggressive fluid hydration. Shortly after treatment initiation, the patient displayed incremental improvements in clinical appearance as well as on interval labs. Unfortunately, the patient's recovery was complicated by worsening tachycardia, tachypnea, and new-onset episodes of hypoxia despite being on prophylactic subcutaneous heparin. CT pulmonary angiography subsequently identified the presence of bilateral submassive pulmonary emboli (PE) as seen in Figure 2.

A course of therapeutic low-molecular-weight heparin was initiated with improvements in hypoxia and the associated symptoms of PE. Repletion with dopaminergic agonists was tapered off concurrently with the reversal of NMS, and the patient returned to his baseline function. The patient transitioned to apixaban anticoagulant for PE and was discharged home to his family. Ultimately, the patient followed up with his private psychiatrist without recurrence of NMS to date, and the therapeutic decision to manage his DMDD and autism with behavioral disturbances was changed accordingly.

## Discussion

Despite their frequent association with first-generation neuroleptics, other medications that act on dopaminergic receptors can also precipitate NMS but are often underdiagnosed due to their milder clinical presentations. These groups of medications include atypical antipsychotics, dopamine-acting antiemetics, and dopaminergic potentiating agents. We have included an unexhaustive list of dopamine-active examples within Table 2 for reference.

**Table 2:** Examples of Dopaminergic Medications Associated With NMS Table representing an unexhaustive categorical list of dopaminergic agents that can be attributable to NMS. (Abbreviations: NMS: neuroleptic malignant syndrome)

Examples of Dopaminergic Medications Associated With NMS	
Typical Antipsychotics	Haloperidol, Pimozide, Trifluoperazine, Fluphenazine, Thioridazine, Chlorpromazine
Atypical Antipsychotics	Olanzapine, Clozapine, Quetiapine, Asenapine, Risperidone, Ziprasidone, Iloperidone, Paliperidone, Lurasidone, Aripiprazole
Antiemetics	Metoclopramide, Promethazine, Prochlorperazine, Droperidol, Domperidone
Dopamine Potentiators	Levodopa, Entacapone, Tolcapone, Amantadine, Pramipexole

**Figure 3:**
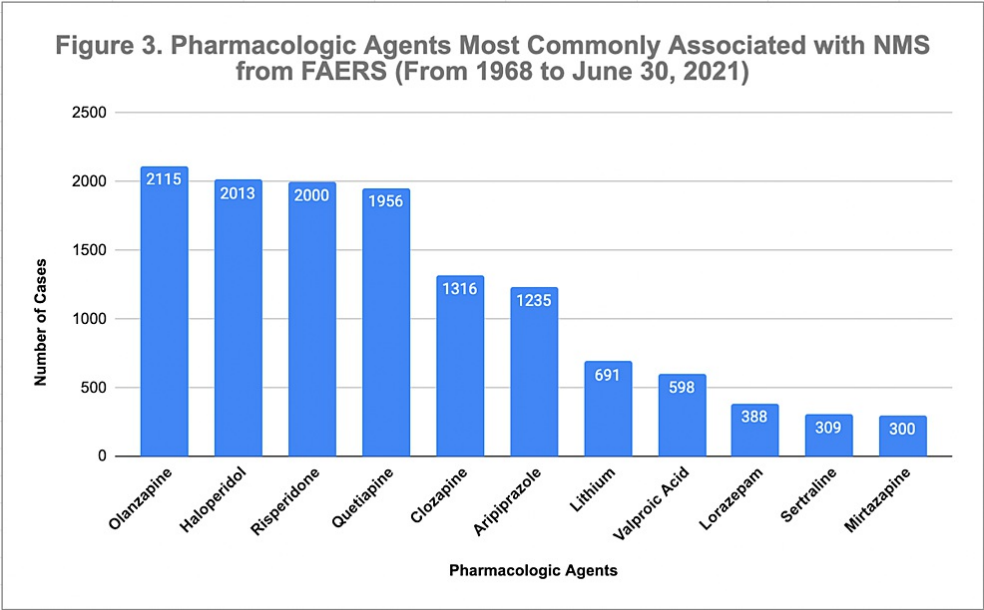
Pharmacologic Agents Most Commonly Associated with NMS from FAERS (From 1968 to June 30, 2021) Figure depicting the most common attributable generic medications to NMS with statistical data collected by the FDA Adverse Event Reporting System (FAERS) from 1968 to June 30, 2021 [2]. The data collection method was done using the following steps on the FAERS website: 1) Searched by reaction term using "Neuroleptic Malignant Syndrome", 2) filtered report of cases by generic names, 3) filter received years to include 1968 to 2021. Of note, the medications are combined under the same generic name for different formulations and administration routes ([olanzapine + olanzapine pamoate]; [haloperidol + haloperidol lactate + haloperidol decanoate]; [quetiapine + quetiapine fumarate]; [aripiprazole + aripiprazole lauroxil]; [lithium + acetate, aspartate, carbonate, citrate, sulfate]; [valproic acid + valproate sodium + divalproex sodium] (Abbreviations: FDA: Food Drug Administration, FAERS: FDA Adverse Event Reporting System; NMS: neuroleptic malignant syndrome) Adapted from [2]

Comprehensively, statistical data retrieval from the Food & Drug Administration (FDA) Adverse Event Reporting System (FAERS) was reviewed for common attributable agents to NMS from June 1968 to October 2021 [2]; of which surprisingly, some of these medications did not have a significant direct effect on dopamine receptors. The data were concisely converted into a bar graph that is depicted in Figure 3.

Notoriously, NMS is represented by the tetrad of high spiking fevers, “lead-pipe”-like rigidity, autonomic instability, and altered mental status, often in the form of agitated delirium or confusion with fluctuation in consciousness. Although this article referred to the Diagnostic and Statistical Manual of Mental Disorders Fifth Edition (DSM-5) and followed the American Psychiatric Association consensus recommendations for diagnosing NMS, it should be recognized that there are several different diagnostic criteria that can broaden the potential for diagnosis. For example, the DSM-5 is notably different in comparison to its previous editions by not secluding a specific diagnostic requirement and is based upon the likelihood of NMS relative to the increasing presence of associated clinical features while excluding any organic etiologies. This arrangement for an absent DSM-5 cut-off criteria has made it more useful in the aspect of being able to unrestrictedly diagnose patients and provide prompt treatment when the symptoms do not fully fulfill a specific diagnostic requirement. The clinical features described within DSM-5 are summarized and can be referenced in Table 3 [3]. Additionally, the inclusion of other popularly referenced criteria can further elaborate on their variations while providing access to diagnostic alternatives. These criteria are chronologically described in Tables 4-7, starting with Levenson’s NMS Criteria of 1985, Adityanjee’s Revised NMS Research Criteria of 1999, DSM-4 Text-Revised Modified NMS Research Criteria of 2000, and the NMS International Expert Consensus Research Criteria of 2011.

**Table 3:** DSM-5 Clinical Features in Diagnosing NMS (2013) Table entailing the DSM-5 clinical features when diagnosing NMS. The likelihood of diagnosis is increased proportionally to the presence of clinical features after the exclusion of medical and mental disorders (Abbreviations: DSM-5: Diagnostic and Statistical Manual of Mental Disorders Fifth Edition; NMS: neuroleptic malignant syndrome) Adapted from [3]

DSM-5 Clinical Features in Diagnosing NMS (2013)
Cardinal Clinical Features for NMS Diagnosis by DSM-5
- Exposure to a dopamine antagonist or dopamine agonist withdrawal within 72 hours of symptom onset
- Generalized muscle rigidity
- Fever > 100.4 F (38.5C) measured orally on at least two interval occasions
Other Associated Clinical Features for NMS Diagnosis by DSM-5
- Neurological Symptoms: tremor, sialorrhea, akinesia, dystonia, trismus, myoclonus, dysarthria, or dysphagia
- Mental Status Changes: characterized by delirium or fluctuating consciousness
- Autonomic Instability: tachycardia (> 25% above baseline), profuse diaphoresis, labile blood pressure (Changes in ≥ 20 mmHg diastolic or ≥ 25 mmHg systolic), urinary incontinence, or pallor.
- Tachypnea and Respiratory Distress: increased respiratory rate > 50% above baseline (possibly due to metabolic acidosis and hypermetabolism, restrictive chest wall from rigidity, aspiration pneumonia, or pulmonary embolism)
- Laboratory Findings: elevated creatinine phosphokinase > 4 times the upper normal limit, leukocytosis, metabolic acidosis, hypoxia, or decreased serum iron.
- Electroencephalogram with generalized slowing.

**Table 4:** Levenson's Criteria for Diagnosing NMS (1985) Table describing Levenson's criteria for diagnosing NMS. It indicates a high probability of NMS in the presence of all three major features or two major and at least four minor findings [4,5]. It should be noted that this criterion does not account for the altered sensorium that is a commonly associated feature of NMS. (Abbreviations: NMS: neuroleptic malignant syndrome) Adapted from [5].

Levenson’s Criteria for Diagnosing NMS (1985)
Major Characteristics
- Fever
- Rigidity
- Elevated creatinine phosphokinase
Minor Characteristics
- Tachycardia
- Tachypnea
- Diaphoresis
- Labile blood pressure
- Leukocytosis

**Table 5:** Adityanjee's Research Criteria for Diagnosing NMS (1999) Table representing Adityanjee’s Research Criteria for diagnosing NMS, which was revised in 1999 from the originally proposed version in 1988. A definitive diagnosis of NMS would require the presence of criteria 1 through 6 being met. The diagnosis of atypical NMS can be made even in the absence of extrapyramidal symptoms listed in criterion 2 if there is at least one associated supportive laboratory finding [5,6]. (Abbreviations: NMS: neuroleptic malignant syndrome) Adapted from [5]

Adityanjee’s Research Criteria for Diagnosing NMS (1999)	
1) Altered sensorium documented by ≥ 2 observers on ≥ 2 consecutive days as confusion, clouding of consciousness, disorientation, mutism, stupor, or coma	
2) Extrapyramidal symptoms (e.g., muscle rigidity, dysphagia, or dystonia)	
3) Fever ≥ 101.3 F (38.5 C) orally for > 48 hours in the absence of medical causes	
4) Autonomic dysregulation (requiring at least two characteristics)	Tachycardia (> 100 heartbeats per minute)
	Tachypnea (> 25 breaths per minute)
	Labile blood pressure (Changes in ≥ 30 mmHg systolic or ≥ 15 mmHg diastolic)
	Urinary incontinence
	Diaphoresis
5) Onset of symptoms with exposure to any of the following:	Dose changes or discontinuation of an antipsychotic drug, dopamine blocker or depleting agent, or psychostimulant drug within 2 weeks.
	Withdrawal of antiparkinsonian or anticholinergic drug during previous 1 week.
	Intramuscular administration of a long-acting antipsychotic depot medication during the past 8 weeks
6) Exclusion of symptoms that are due to any existing or new-onset medical, neurological, or psychiatric disorders (secondary to substance abuse, infectious illnesses, metabolic, delirium, encephalitis, epilepsy, brain tumors, catatonic schizophrenia, mood disorder with catatonic features, etc.)	
7) Supportive laboratory features (any one of the following)	Leukocytosis
	Elevated transaminases (liver dysfunction enzymes)
	Myoglobinuria
	Elevated creatinine phosphokinase
	Low serum iron

**Table 6:** Modified DSM-4-TR Research Criteria for NMS (2000) Table depicting all the criteria that must be met to establish a definitive diagnosis of NMS set forth by the DSM-4-TR. It is largely recognized by its inclusive sensitivity of 69.6% and relatively high specificity of 90.7% [7,8]. (Abbreviation: DSM-4-TR: Diagnostic and Statistical Manual of Mental Disorders Fourth Edition Text-Revised; NMS: neuroleptic malignant syndrome) Adapted from [7].

Modified DSM-4-TR Research Criteria for NMS (2000)	
1) Development of muscle rigidity and elevated temperature associated with the use of neuroleptic medication.	
2) Two or more of the following symptoms:	Diaphoresis
	Dysphagia
	Tremor
	Incontinence
	Changes in level of consciousness ranging from confusion to coma
	Mutism
	Tachycardia
	Elevated or labile blood pressure
	Leukocytosis
	Laboratory evidence of muscle injury (e.g., elevated creatinine phosphokinase)
3) Symptoms of criteria 1 and 2 not attributable to illicit drug use, neurological, or medical condition	
4) Symptoms of criteria 1 and 2 are not accountable to a mental disorder	

**Table 7:** International Expert Consensus Study for Diagnosing NMS (2011) Table representing the most modernly proposed diagnostic criteria for NMS developed from the International Consensus Study. This criterion uses a point system in the presence of each clinical feature and laboratory findings. A cutoff of 74 points out of the 100 points total is considered a high probability for NMS [8,9]. (Abbreviations: NMS: neuroleptic malignant syndrome) Adapted from [8]

International Expert Consensus Study for Diagnosing NMS (2011)	
Exposure to dopamine antagonist or withdrawal of dopamine agonist within 72 hours (*20 points*)	
Fever ≥ 100.4 F (38 C) measured orally on two different occasions (*18 points*)	
Muscle Rigidity (*17 points*)	
Altered Mental Status (*13 points)*	
Elevated creatinine phosphokinase ≥ 4 times the upper limit of normal (*10 points*)	
Sympathetic lability, requiring at least 2 or more of the following: (*10 points*)	Elevated blood pressure (Systolic or diastolic pressures ≥ 25% above baseline)
	Blood pressure fluctuation (A change of 25mmHg systolic or 20mmHg diastolic pressures within 24 hours)
	Urinary incontinence
	Diaphoresis
Negative workup for infectious, metabolic, neurologic, or toxic etiologies (*7 points*)	
Tachycardia (≥ 25% above baseline) and tachypnea (≥ 50% above baseline) (*5 points*)	

**Figure 4:**
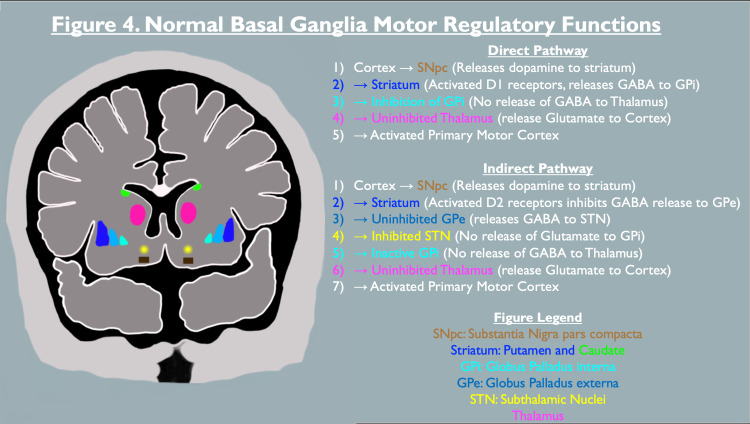
Basal Ganglia Motor Regulatory Functions Figure illustrating the normal pathway of dopaminergic potentiation upon the basal ganglia leading to modulation of cortical motor activity. A deprivation in D2 activity will lead to overt activation of the indirect inhibitory pathway causing decreased movement, involuntary hypertonia, and rigid muscle. Synergistically, a lack of D1 activity in the direct excitatory pathway will also promote decreased movement and rigidity. The cortical structures are represented by color-coded locations and the included figure legends identify the structures accordingly. (Abbreviations: SNpc: substantia nigra pars compacta, GPi: globus pallidus interna, GPe: globus pallidus externa, STN: subthalamic nuclei, GABA: gamma-aminobutyric acid, D1: dopamine-1 receptor, D2: dopamine-2 receptor)

The pathophysiology of NMS is not completely understood but is theoretically thought to revolve around an abrupt decrease or cessation in centrally acting dopaminergic activity. This central dopamine hypoactivity theory considers D2 blockade within the hypothalamus as a potential cause of hyperthermia and autonomic hyperstimulation. Furthermore, D2 receptor activation within the basal ganglia is known to regulate and cause an inhibitory effect on the indirect motor regulatory pathway as represented in Figure 4. Therefore, a decrease in D2 activity will result in a decreased propensity for movement and provides an acceptable explanation for muscle rigidity and Parkinsonian-like symptoms. Trivially, due to the presumptive pathophysiology of NMS being identical to akinetic Parkinsonian crisis, these two disorders were historically designated synonymously as malignant dopamine depletion syndrome [10].

Even though NMS is frequently recognized by its linkage to antipsychotic medications, the reported rate of NMS attributable to antipsychotics is declining. This is likely due to increasing mandatory prescribing precautions (e.g., FDA black box warnings, hard-stops in electronic prescribing databases), increasing adversity awareness, and implementation of newer generations of antipsychotics, preferentially for their fewer and milder side effects [9]. Diverging from dopamine, our unusual case of NMS occurred in the absence of antipsychotic inclusion and posed as a major diagnostic dilemma. Currently, the scientific literature is sparse, but there have been cases of NMS that are recognized with the use of lithium, benzodiazepines, and other neuromodulators [11]. As a result, clinicians should consider and be aware of other centrally active pharmacologic agents and neurotransmitters that may potentiate NMS (e.g., gamma-aminobutyric acid, acetylcholine, serotonin, norepinephrine, and epinephrine). As more cases of NMS arise in the absence of antipsychotics or medications that directly alters the functional availability of dopamine, further research is necessary to identify other potential causes of NMS, especially if these added culprits can implicate diagnostic criteria revisions to ensure prompt diagnosis and treatment.

Due to the timeframe of the abrupt medication stoppage and the absence of direct antidopaminergic agents in this patient, we hypothesized the etiology of this patient’s NMS is most likely triggered by the unintentional withdrawal of oxcarbazepine and topiramate. From a review of the current literature, oxcarbazepine and topiramate are well-recognized to promote and indirectly increase the release of dopamine within the central nervous system [12-14]. During sudden oxcarbazepine and topiramate cessation, this would have led to a decrease in dopamine released and potentiated the risk for a hypodopaminergic state to create this rare NMS presentation. Additionally, dysfunction of other neurotransmitters should also be recognized in triggering the autonomic and neurological features of this disorder. Specifically, glutamate hyperexcitation has been noted in oxcarbazepine and topiramate withdrawal as a rebound effect from their mechanism of action [15,16]. Glutamate neurotoxicity is also well-established in the setting of NMS within the basal ganglia [17]. Addressing this glutamic acid hyperexcitation problem could prevent further destructive cascade on other neurotransmitters and therefore treatment with N-methyl-D-aspartate (NMDA) receptor antagonists, such as amantadine and memantine, has been advocated for their effectiveness in reducing the glutamate surge as well as increasing dopamine release [18].

Importantly, this case also highlights the emphasis on avoiding any psychotropic medication when facing clinical doubt for the diagnosis of NMS, as it is critical to avoid worsening detrimental morbidities and possible death. Following recognition and diagnosis of NMS, an aggressive treatment plan should be promptly initiated to prevent further clinical deterioration. Giving consideration to identifying the severity of the NMS presentation can directly correspond to a stepwise approach in pharmacologic treatments. Synergistically, pharmacotherapy can be supplemented with electroconvulsive therapy (ECT) when encountering moderate to severe NMS cases that are rapidly worsening or refractory to medication. Furthermore, ECT is relatively safe and requires six to 10 sessions for treating acute NMS, however, the cautious use of succinylcholine during peri-procedural anesthesia should be carefully contemplated in patients with severe rhabdomyolysis to avoid accentuating hyperkalemia and the risk of cardiovascular complications [19,20]. Various treatment plans can be formulated based on the patient's stability and clinicians’ preferences; however, we objectively referenced the Woodbury severity stages of NMS in combination with the corresponding evidence-based management approach elaborated in Table 8 [20-23].

**Table 8:** Treatments Based on Severity of NMS Symptoms Using the Woodbury Staging System Table incorporating the Woodbury stages of NMS symptoms in combination with the corresponding treatment approach. The first-line therapy is always supportive care for the patients’ clinical presentation and is followed by prompt pharmacologic treatment escalation as needed to prevent further worsening of symptoms. Adjunctive electroconvulsive therapy can be added in the more severe cases of rigidity with rapid clinical deterioration [20-23]. It should also be noted that any orally administered medications can also be given via nasogastric or percutaneous gastrostomy tube if the formulation is available. (Abbreviations: PO: per os/orally, IM: intramuscular, IV: intravenous, ECT: electroconvulsive therapy; NMS: neuroleptic malignant syndrome) Adapted from [21].

Treatment Algorithm for NMS Symptoms Using the Woodbury Staging System			
Woodbury Stages	Clinical Features	Supportive Care	Pharmacologic and Adjunctive Treatment
Stage 1: drug-induced parkinsonism	Rigidity, tremor	Reduce or switch antipsychotics	Anticholinergic agents
Stage 2: drug-induced catatonia	Rigidity, mutism, stupor	Stop, reduce, or switch antipsychotics	Lorazepam: 1-2mg IM/IV q4-6 hrs.
Stage 3: mild, early NMS	Mild rigidity, catatonia or confusion, temperature ≤ 38℃ (100.4℉), heart rate ≤ 100	Stop antipsychotics, monitor for progression with symptomatic care (e.g., electrolytes, fluid)	Lorazepam: 1-2mg IM/IV q4-6 hrs.
Stage 4: moderate NMS	Moderate rigidity, catatonia or confusion, temperature 38-40℃ (100.4-104℉), heart rate 100-120	Stop antipsychotics, upgrade to intensive care with cooling measures, and manage symptoms & complications	Lorazepam: 1-2mg IM/IV q4-6 hrs. PLUS Bromocriptine: 2.5-5mg PO q8 hrs. OR Amantadine: 100mg PO q8 hrs. Adjunct therapy: Consider ECT for 6-10 bilateral treatments
Stage 5: severe NMS	Severe rigidity, catatonia or coma, temperature ≥40℃ (104℉), heart rate ≥ 120	Stop antipsychotics, upgrade to intensive care with cooling measures, and manage symptoms & complications	Lorazepam: 1-2mg IM/IV q4-6 hrs. Dantrolene: 1-2.5mg/kg IV q6 hrs. for 48 hours then taper with PO Bromocriptine: 2.5-5mg PO q8 hrs. Amantadine: 100mg PO q8 hrs. Adjunct therapy: Consider ECT for 6-10 bilateral treatments

Acknowledgment of the complications within this case can be appreciated to heighten our vigilance for future encounters. One valid concern is difficulty in confirming muscle rigidity and stiffness in a patient that is highly vulnerable to being overlooked as possible secondary responses to underlying diagnoses (i.e., this patient’s developmental delays, behavior issues, and spastic cerebral palsy). An argument can be made that this patient could be rigidly tensed from his spastic cerebral palsy, lingering neurological deficit, or behaviorally hypervigilant or defiant with unfamiliarized medical staff and hospital settings. Hence reasonably, when encountering patients with stiffness along with a variety of comorbidities, physicians should keep a high level of suspicion for abnormal presentations and include a broad differential diagnosis. This patient’s case was also suspected to have intracranial pathology, infectious meningoencephalitis, lethal catatonia, depression with psychosis, seizures, and malignant hyperthermia as possible differential diagnoses. In Table 9, we have included a non-exhaustive summarization of other probable differential diagnoses when attempting to rule out NMS [20].

**Table 9:** Differential Diagnosis of NMS Table recognizing some of the common diagnoses that should be considered when ruling out NMS. However, this is only an unexhausted differential diagnosis list when considering presentations similar to NMS. (Abbreviations: NMS: neuroleptic malignant syndrome) Adapted from [20].

Differential Diagnosis of NMS	
Infectious Diseases	CNS infections (e.g., meningitis or encephalitis)
	Brain abscess
	Progressive multifocal leukoencephalopathy
	Neurocysticercosis
	Post-infectious encephalomyelitis syndrome
	Tetanus
	Severe sepsis from systemic infections (e.g., pneumonia, catheter-related bloodstream infection)
Neuropsychiatric	Malignant catatonia
	Agitated delirium
	Medication-induced extrapyramidal side effects
	Tonic seizure or nonconvulsive status epilepticus
	Acute spinal cord injury
	Subdural hematoma
	Structural brain lesions, particularly involving the midbrain
	Autoimmune encephalitis
Toxic overdose and poisoning or Medication-induced	Anticholinergic delirium
	Malignant hyperthermia
	Salicylate poisoning
	Serotonin syndrome
	Intoxication influenced by Substances of Abuse (e.g., cocaine, ecstasy, amphetamines)
	Withdrawal from dopamine agonists, baclofen, sedative-hypnotics, and alcohol
Endocrine	Thyrotoxicosis
	Pheochromocytoma
	Paraganglioma
Environmental	Heat stroke

Moreover, this patient suffered from extensive bilateral pulmonary embolisms that are likely provoked by NMS, even in the presence of thromboprophylaxis with subcutaneous heparin. Physicians should pay extra attention to the immobilization effects in NMS because of their high risk for developing venous thromboembolism. Several case studies have shown the development of deep vein thrombosis and pulmonary embolisms despite pharmacologic thromboprophylaxis and therefore early mobilization with assistance should be attempted whenever possible [24-26].

## Conclusions

This case summarizes the pathophysiology of NMS and emphasizes the importance of identifying other unfamiliar etiologies when antipsychotics are not in use. It described a rarely encountered presentation of NMS following abrupt withdrawal from topiramate and oxcarbazepine. This article also reiterates the need for early treatment to prevent further complications and therefore in highly suspicious circumstances, clinicians should be aware of other possible causes of NMS.
